# Focus on the Mental Health of Pediatric Medical Workers in China After the COVID-19 Epidemic

**DOI:** 10.3389/fpsyg.2021.657814

**Published:** 2021-04-20

**Authors:** Hui Liu, Li Wang

**Affiliations:** ^1^Department of Pediatrics, University-Town Hospital of Chongqing Medical University, Chongqing, China; ^2^Department of Pediatrics, Daping Hospital, Army Medical University, Chongqing, China

**Keywords:** mental health, pediatric medical workers, COVID-19, epidemic, China

## Abstract

As was previously known, pediatric medical staff in China faced several hurdles including high occupational risk, multiple contradictions, heavy workload, and long working hours. After the outbreak of 2019 novel coronavirus, facing the overload of work and the potential risk of infection, pediatric medical workers may be under great psychological pressure. The purpose of this article was to call attention to the impact of the epidemic on the mental health of Chinese pediatric workers, and developing psychological intervention program that are tailored to them. The experiences from this public health emergency should inform the efficiency and quality of future crisis intervention of the Chinese government and authorities around the world.

## Introduction

The outbreak of the coronavirus disease 2019 (COVID-19) that started in Wuhan, China, in December 2019, quickly spread across the whole country and has attracted worldwide attention. To quickly control the epidemic and save the lives of infected patients, Chinese medical workers have been extremely busy working hard over the past 1 year and have made great sacrifices. However, according to incomplete statistics, up to now, more than 3,380 medical staff from 476 medical institutions in China have been infected with novel coronavirus [Bureau for Disease Control and Prevention (BDCP), 2020].

Due to the dangerous epidemic situation, medical resources were once very tight. Facing overwork, frustration, isolation, a lack of contact with their families and other stressors, medical staff have been exhausted and borne enormous pressure, most of them have experienced anxiety, depression, insomnia, denial, anger, fear, and other related negative emotions during the epidemic (Kang et al., 2020a). Therefore, psychological crisis intervention has become another important task in the fight against COVID-19. The Chinese government incorporated psychological crisis intervention into the overall efforts for epidemic prevention and control, and the National Health Commission of China published a national guideline for psychological crisis intervention for COVID-19 on January 27, 2020 [National Health Commission of the People’s Republic of China (NHCPRC), 2020]. However, the psychological intervention for pediatric medical staff has not attracted the attention of the government and relevant departments because of the relatively low incidence in children. Thus far, there is no relevant report on the protective measures for the mental health of pediatric medical workers.

Here, we list three important reasons for calling on the relevant departments to pay attention to the psychological intervention of pediatric medical workers during and after the epidemic. First, according to the “White Paper on The Current Situation of Pediatric Resources in China,” issued by the pediatric branch of Chinese Medical Association [Pediatric Branch of Chinese Medical Association [PBCMA], and Paediatric Branch of Chinese Medical Doctor Association [PBCMDA], 2017], the number of pediatric medical staff in China is seriously insufficient, one pediatrician must serve more than 2,000 children (Li et al., 2017), and they usually are associated with high occupational risk, conflicts between doctors and patients, heavy workload, long working hours, low pay, and other negative factors. It can be seen that Chinese pediatric medical staff are under greater mental pressure than those in other developed countries. These pediatric staff members have suffered a higher incidence of physical violence and psychological pressure than other Chinese medical workers (Li et al., 2017). Second, respiratory disease is one of the most common pediatric diseases, 73.11% of pediatric outpatients in China are inclined to respiratory disease (Xiong et al., 2017). However, it is very difficult to distinguish COVID-19 from common respiratory disease in the early stage. With students gradually returning to school, the chance of cross-infection among children increased. As a result, common respiratory disease showed a small outbreak trend in the early stage of returning to school. Furthermore, parents expressed more anxiety and panic than usual once children had fever, cough, and other symptoms. A single center study in China showed that 25.7% of parents in the pediatric outpatient had anxiety symptoms, especially, women and people over the age of 50 showed higher anxiety (34.8 and 54.1%, respectively) (Li and Wu, 2021). Although the morbidity of COVID-19 in children was not high, the above behaviors indirectly brought an increased workload, a high risk of infection and psychological distress to pediatric medical workers who bore new psychological pressure again. Third, it was reported that the health-related quality of life of pediatric medical workers declined during the COVID-19 epidemic (Huang et al., 2020), 10.3% of respondents represented moderate or severe psychological impact, and 4.0% showed severe anxiety symptoms (Zhang et al., 2020), which was higher than the general Chinese population prevalence of severe anxiety symptoms (2.3%) during the epidemic (Wang S. et al., 2020). Also, our investigation (Liu et al., 2020) found that depression (14.8%) and anxiety (18.3%) were present to varying degrees among pediatric medical workers across the country. By contrast, 11.0 and 12.2% of participants had depression symptoms and anxiety symptoms, respectively in the general Chinese population during the COVID-19 epidemic (Wang S. et al., 2020). Moreover, the rate of depression in pediatric medical staff (14.8%) was even higher than that in general medical staff (12.10%) (Liu et al., 2020; Lu et al., 2020).

In conclusion, timely psychological intervention for pediatric medical workers is very urgently needed. Although victory has been declared against the initial stage of the COVID-19 epidemic in China, the psychological stress and trauma suffered by pediatric medical staff in this epidemic will not disappear immediately with the end of the epidemic. Those who performed epidemic-related tasks are at risk of experiencing posttraumatic stress disorder symptoms, which have been proved in similar international outbreaks in recent years, such as severe acute respiratory syndrome (SARS) in 2003 (Chong et al., 2004), Ebola virus disease (EVD) in 2014 (Shultz et al., 2015), and Middle East respiratory syndrome coronavirus (MERS-CoV) in 2015 (Lee et al., 2018). Therefore, we call for the relevant departments to carry out extensive research on the psychological status of pediatric medical workers, as well as targeted psychological interventions, which mainly cover the following several areas. First, formulating psychological intervention guidelines for below high-risk pediatric medical workers, and carrying out targeted psychological intervention. According to the previous studies, those who had high education (master and above), senior titles or aged between 30 years old and 60 years old were prone to psychological problems (Huang et al., 2020; Liu et al., 2020). Compared with nurses, doctors suffered from more stress due to first physical examination and medical decisions (Huang et al., 2020). Meanwhile, those who had been exposed to confirmed or suspected COVID-19 patients or worked in Hubei province faced a greater psychological burden (Huang et al., 2020; Liu et al., 2020). These pediatric medical staff whose hospitals did not have fever clinics and isolated observation areas experienced lower health-related quality of life (Huang et al., 2020). Second, a psychological intervention medical team should be built to provide face-to-face psychological counseling and various group activities for medical workers with moderate and severe mental disorders, who than those with subthreshold and mild mental distress are more eager to receive one-on-one assistance or group psychotherapy from psychologists or psychiatrists (Kang et al., 2020b). Third, psychological interventions should provide various psychological self-help brochures or media publicity to release stress. A study found that medical staff with subthreshold and mild psychological disturbances preferred to these methods to rescue themselves, and were willing to use these skills to help others (Kang et al., 2020b), which have been proven to be beneficial to their later mental health (Maunder et al., 2006). Fourth, it is necessary to establish a psychological assistance hotline and online mental health services which could provide guidance and supervision to solve psychological problems. Online consulting is an effective way to reduce the risk of face-to-face contact because of providing initial screening for those who need face-to-face counseling, and is applicable to medical staff of various departments (Geoffroy et al., 2020). Fifth, training on the knowledge of protective measures against COVID-19 can be arranged for parents and children to relieve their anxiety and prevent COVID-19 infection. Some studies showed that the mastering of preventive measures (e.g., wearing masks, hand hygiene) was related to lower levels of stress, anxiety and depression (Wang C. et al., 2020), meanwhile, the understanding of knowledge of COVID-19 was associated with reduction of psychological disorders (Galić et al., 2020). Sixth, online medical services and outpatient appointment systems should be optimized to alleviate the aggregation of pediatric outpatients. During the peak of the COVID-19 epidemic, online medical services were feasible for pediatric rehabilitation and non-emergency pediatric patients, which could reduce the risk of cross-infection and the waste of medical resources (Tanner et al., 2020; Yang et al., 2021), and online appointment systems could save patients’ time and improve patients’ satisfaction (Cao et al., 2011). Seventh, hospital security staff can be made available to be sent to help deal with uncooperative patients. A study from a pediatric outpatient in China found that some parents refused their children to accept nucleic acid testing (10.39%) and transfer to fever clinic (1.17%), asked for earlier access (4.43%) (Li and Wu, 2021), which increased the incidence of conflicts between doctors and patients. These measures to reduce the psychological pressure of pediatric medical staff would have a profound impact on the fight against the epidemic and partly alleviate the current situation of pediatric medical resources shortage in China. The experiences from this public health emergency should inform the efficiency and quality of future crisis intervention by the Chinese government and authorities around the world.

## Data Availability Statement

The original contributions presented in the study are included in the article/supplementary material, further inquiries can be directed to the corresponding author/s.

## Author Contributions

Both authors conceptualized and designed the study, drafted the manuscript, and approved the final manuscript as submitted.

## Conflict of Interest

The authors declare that the research was conducted in the absence of any commercial or financial relationships that could be construed as a potential conflict of interest.
